# *Bacillus subtilis* RarA Acts as a Positive RecA Accessory Protein

**DOI:** 10.3389/fmicb.2020.00092

**Published:** 2020-02-13

**Authors:** Hector Romero, Ester Serrano, Rogelio Hernández-Tamayo, Begoña Carrasco, Paula P. Cárdenas, Silvia Ayora, Peter L. Graumann, Juan C. Alonso

**Affiliations:** ^1^Department of Microbial Biotechnology, Centro Nacional de Biotecnología, CSIC, Madrid, Spain; ^2^SYNMIKRO, LOEWE-Zentrum für Synthetische Mikrobiologie, Marburg, Germany; ^3^Fachbereich Chemie, Philipps-Universität Marburg, Marburg, Germany

**Keywords:** Mgs1, WRNIP1, replication stress, RecA mediators, RecA modulators

## Abstract

Ubiquitous RarA AAA^+^ ATPases play crucial roles in the cellular response to blocked replication forks in pro- and eukaryotes. Here, we provide evidence that absence of RarA reduced the viability of Δ*recA*, Δ*recO*, and *recF*15 cells during unperturbed growth. The *rarA* gene was epistatic to *recO* and *recF* genes in response to H_2_O_2_- or MMS-induced DNA damage. Conversely, the inactivation of *rarA* partially suppressed the HR defect of mutants lacking end-resection (Δ*addAB*, Δ*recJ*, Δ*recQ*, Δ*recS*) or branch migration (Δ*ruvAB*, Δ*recG*, Δ*radA*) activity. RarA contributes to RecA thread formation, that are thought to be the active forms of RecA during homology search. The absence of RarA reduced RecA accumulation, and the formation of visible RecA threads *in vivo* upon DNA damage. When Δ*rarA* was combined with mutations in genuine RecA accessory genes, RecA accumulation was further reduced in Δ*rarA* Δ*recU* and Δ*rarA* Δ*recX* double mutant cells, and was blocked in Δ*rarA recF*15 cells. These results suggest that RarA contributes to the assembly of RecA nucleoprotein filaments onto single-stranded DNA, and possibly antagonizes RecA filament disassembly.

## Introduction

During DNA replication, the replisomes encounter obstacles that can block their progression, and replication impairment is recognized as an important source of genetic instability (Kuzminov, 1995; Haber, 2015; Gaillard and Aguilera, 2016). Maintenance of genome stability is one of the crucial functions in life. As a consequence, numerous and diverse mechanisms have evolved to minimize the frequency or impact of replicative stress (Kuzminov, 1995; Mirkin and Mirkin, 2007; Branzei and Foiani, 2008; Gaillard and Aguilera, 2016). Eukaryotic Mgs1/WRNIP1 and prokaryotic RarA, which are evolutionarily conserved AAA^+^ ATPases associated with a variety of cellular activities, play important but poorly understood roles in cellular responses to stalled or collapsed replication forks (Barre et al., 2001; Hishida et al., 2001, 2002, 2006; Shibata et al., 2005; Tsurimoto et al., 2005; Saugar et al., 2012; Leuzzi et al., 2016; Stanage et al., 2017; Carrasco et al., 2018; Romero et al., 2019a, b).

Previous assays have indicated a poorly understood role for bacterial RarA in homologous recombination (HR). A *Bacillus subtilis* null *rarA* (Δ*rarA*) mutant strain renders cells very sensitive to H_2_O_2_, but not to methyl methane sulfonate (MMS) or to UV radiation-mimetic compound 4-nitroquinoline-1-oxide (Romero et al., 2019b). In contrast, an *Escherichia coli* Δ*rarA* strain remains as capable of repairing UV-induced DNA damage as wild-type (*wt* or *rec*^+^) cells (Barre et al., 2001; Shibata et al., 2005). In both bacteria, *E. coli* and *B. subtilis*, the viability under unperturbed conditions of Δ*rarA* Δ*recA* cells is significantly lower than that of the Δ*recA* control (Shibata et al., 2005; Romero et al., 2019b). Since the *recA* gene is not epistatic with functions involved in base or nucleotide excision repair, but the *E. coli* or *B. subtilis rarA* gene is epistatic to *recA* in response to DNA damage (Shibata et al., 2005; Romero et al., 2019b), we assume that RarA is a genuine repair-by-recombination protein.

Bacterial RarA shares structural similarity with DnaX, a subunit of the clamp loader complex (Page et al., 2011), but *B. subtilis* RarA could not substitute for DnaX in the cognate reconstituted *in vitro* DNA replication system (Carrasco et al., 2018). Rather, these assays showed that RarA, together with its interacting partner SsbA, inhibited initiation of PriA-dependent DNA replication, but not chain elongation, suggesting that RarA might impede the assembly of the replicative helicase and prevent that recombination intermediates contribute to pathological DNA replication restart (Carrasco et al., 2018). In addition to RarA, SsbA also interacts with various recombination (RecQ, RecS, RecJ, RecG, RecO, RecD2, SbcC, and SbcE) and replication (PriA, DnaG, and DnaE) proteins, of which RecS, RecD2, SbcE, and DnaE are absent in *E. coli* cells (Costes et al., 2010). These data suggest a role of RarA in recombination-dependent DNA replication, although RarA might follow different avenues in distantly related bacteria or depending on the type of DNA damage (Stanage et al., 2017; Carrasco et al., 2018; Romero et al., 2019a, b). For example, when DNA replication is blocked, upon dNTPs depletion by hydroxyurea, RarA*_*Eco*_* foci disassemble from the replication fork and disappear *in vivo* (Sherratt et al., 2004). However, *in vitro* studies suggested that RarA*_*Eco*_* may contribute to replication fork rescue by creating a flap on the lagging strand, so that the replicative helicase and its associated replisome could continue chain elongation without the need for replisome disassembly and replication restart (Stanage et al., 2017). In *B. subtilis* cells, inhibition of the replicative DNA polymerase PolC, by the specific inhibitor *p*-hydroxyphenylazo-uracil (HPUra), confines the RarA molecules toward the collapsed replication forks *in vivo* (Romero et al., 2019b). In this bacterium it was shown that *B. subtilis* RarA-mVenus (RarA-YFP) transiently colocalizes with the DnaX-CFP protein, and it alternates between static and dynamic states. RarA-mVenus is confined to the replication forks when the preprimosomal DnaB protein (absent in *E. coli*) is non-functional, but the opposite occurs upon inactivation of the replicative DNA helicase DnaC (counterpart of *DnaB*_*Eco*_) (Romero et al., 2019a, b), revealing an intricate function for this protein related to DNA replication restart.

*B. subtilis* RarA-mVenus forms mobile foci, usually one per cell containing many molecules, that move in a time scale of minutes in ∼50% of total cells, mostly close to replication forks, in which RarA is likely DNA-bound. On a time scale of milliseconds, ∼50% of RarA molecules move very slowly or are static, likely within the slowly moving foci, while the remaining fraction is highly dynamic, diffusing throughout the cells (Hernández-Tamayo and Graumann, 2019; Romero et al., 2019a). DNA damages changed the ratio of static (DNA-bound) and freely diffusive RarA, e.g., H_2_O_2_ decreased the static subpopulation of RarA at the replication forks, and instead, RarA was recruited to areas located away from the replication forks. Exposure to H_2_O_2_ increased the fraction of dynamic molecules, but not treatment with MMS, and this was exacerbated by the absence of end resection or Holliday junction (HJ) processing proteins (Romero et al., 2019a). The number of cells containing slowly moving RarA foci was also affected by several proteins acting in HR (Romero et al., 2019a), indicating that the number of molecules acting within the foci, and the positioning of the foci, is affected by interactions with HR proteins.

**FIGURE 1:**
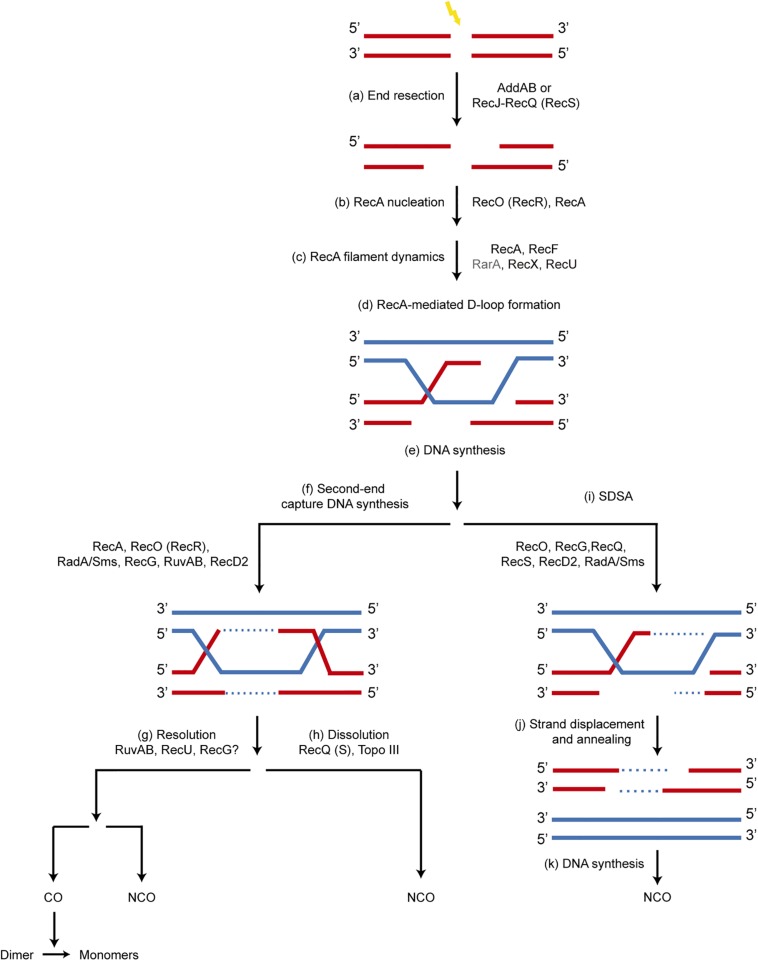
Mechanism of recombinational repair of DSBs in *B. subtilis*. The main players in each step are shown. Since the SsbA protein influences all processes involving ssDNA, it is not depicted for clarity. **(a)** the 5′-ends are resected to produce a 3′-tailed duplex by AddAB or by RecJ in concert with a RecQ-like enzyme (RecQ or RecS). SsbA, which interacts with RecJ, RecQ and RecS, binds to the ssDNA region. **(b)** A set of accessory proteins (RecA mediators, RecO and RecR) act before homology search and contribute to load RecA onto ssDNA displacing bound SsbA, which interacts with RecO. **(c)** The accessory proteins, known as modulators (RecF, RecX, RecU and RarA [this work]) act during homology search and DNA strand exchange, regulating the formation of dynamic RecA filaments (SsbA interacts with RarA, and RecA with RecU and RecX). **(d,e)** Strand invasion of the 3′-end of the invading strand results in a D-loop recombination intermediate and provides the primer for DNA synthesis. **(f)** A capture of the second DNA derived from the other end of the DSB by RecA, RecO, RecR, leads to a double HJ that can be processed by branch migration translocases RecG, RuvAB, RadA/Sms, and may be RecD2. **(g)** RuvAB in concert with RecU resolves the HJ, leading to CO or NCO products. The CO products lead to dimers that are resolved to monomers by specialized site-specific recombinases. **(h)** A type I Topoisomerase in concert with a RecQ-like helicase (RecQ, RecS) may dissolve the double HJ, producing only NCO products. **(i–k)** A synthesis dependent strand annealing mechanism (SDSA) is depicted. The 3′-invading end is extended by the replicase. Then it may be displaced from the joint molecule by branch migration translocases (RuvAB, RecG, RadA/Sms, RecD2, RecQ, RecS) and re-annealed with the complementary strand of the other resected end of the break by the strand annealing activity of RecO, leading to NCO products.

To analyze the role of RarA in repair-by-recombination at the genetic level, the Δ*rarA* deletion was moved into *rec*-deficient strains impaired in DNA end resection (*addAB*, *recQ*, *recS*, *recJ*), RecA mediators (*recO*) and/or modulators (*recF*, *recX*, *recU*), or HJ processing and cleavage/dissolution (*recG*, *ruvAB*, *radA*, *recD*2, *recU*, *recQ*, *recS*) (see Figure 1) and the resulting strains were genetically analyzed. We show that lack of RarA reduces cell viability in the Δ*recO* and Δ*recA* and in less extent in the *recF15* context in the absence of DNA damage, but these single and double mutant strains are equally sensitive to H_2_O_2_- or MMS-induced DNA lesions (epistasis). The absence of RarA partially suppressed the DNA repair defect of cells impaired in DNA end resection (*addAB*, *recQ*, *recS*, *recJ*), or HJ processing and cleavage/dissolution (*recG*, *ruvAB*, *radA*, *recD*2, *recU*, *recQ*, *recS*), suggesting that an alternative pathway(s), inhibited when RarA is present in the cell, may contribute to remove/circumvent the damaged template bases. Lack of RarA may reduce the accumulation of the signal (RecA filament formation) that facilitates LexA self-cleavage and SOS induction, as judged by the drop of RecA levels upon exposure to increasing mitomycin C (MMC) concentrations and the reduced number of RecA threads in Δ*rarA* cells. Together, these data suggest that RarA may facilitate RecA filament growth and might counteract negative mediators RecX and/or RecU.

## Materials and Methods

### Bacterial Strains

*Bacillus subtilis* BG214 and its isogenic derivatives are listed in Supplementary Table S1. The null *rarA* (Δ*rarA*) mutation was transferred into the other genetic backgrounds by SPP1-mediated chromosomal transduction. The *recF*15 point mutation and a null mutation in *recF* (Δ*recF*) are equally deficient in DNA repair, but the latter shows a reduced cell fitness, because it compromises expression of the downstream essential *gyrB* and *gyrA* genes, thus we worked with the inactive *recF*15 strain (Alonso and Stiege, 1991). In RecF15 the highly conserved negatively charged residue E255 is replaced by a positively charged one K255, RecF E255K, rendering an inactive protein (Alonso and Stiege, 1991). The accuracy of the double mutations was analyzed by PCR amplification and nucleotide sequence analyses. Unless otherwise stated, the indicated genes and products are of *B. subtilis* origin.

### Survival Studies

H_2_O_2_, MMS and MMC were obtained from Sigma Aldrich (Germany). The sensitivity of cells to acute exposure to MMS or H_2_O_2_ was determined by growing *rec*^+^ and its isogenic derivative strains (see Tables 1, 2) in NB to an OD_560_ = 0.4 at 37°C with agitation. Then, cells were incubated with increasing concentrations of MMS or H_2_O_2_ for 15 min. Treated cells were diluted and plated on nutrient broth (NB) agar plates, incubated overnight (ON) at 37°C, and the colonies forming units/ml (CFUs/ml) were counted. The large majority of cells were one and two non-separated with an average of ∼1.6 cells/CFU, thus we have assumed an acceptable correlation of OD_560_ with CFUs.

**TABLE 1:** LD_99_ to H_2_O_2_ and MMS of different *Bacillus subtilis* mutant strains.

Relevant genotype	LD_99_ to H_2_O_2_^a^ in mM	Relevant genotype	LD_99_ to H_2_O_2_^a^ in mM	Impairment
*rec*^+^	>6.0	Δ*rarA*	0.38	

Δ*addAB*	0.46	Δ*addAB* Δ*rarA*	4.5	
Δ*recJ*	4.3	Δ*recJ* Δ*rarA*	0.47	End
Δ*recQ*	2.4	Δ*recQ* Δ*rarA*	1.9	Resection
Δ*recS*	4.4	Δ*recS* Δ*rarA*	2.0	

Δ*recU*	0.45	Δ*recU* Δ*rarA*	0.47	
Δ*recG*	0.44	Δ*recG* Δ*rarA*	0.53	Processing
Δ*ruvAB*	0.64	Δ*ruvAB* Δ*rarA*	1.0	Recombination
Δ*radA*	2.0	Δ*radA* Δ*rarA*	4.7	Intermediates

Δ*recO*	0.37	Δ*recO* Δ*rarA*	0.37	RecA
*recF15*	0.37	*recF15* Δ*rarA*	0.37	Accessory
Δ*recX*	0.8	Δ*recX* Δ*rarA*	0.40	Proteins

Δ*recD2*	1.9	Δ*recD2* Δ*rarA*	0.52	Undefined

*^*a*^The acute lethal dose of H_2_O_2_ that reduced cells survival by 99% (LD_99_) upon 15 min exposure.*

**TABLE 2:** LD_99_ to H_2_O_2_ and MMS of different *Bacillus subtilis* mutant strains.

Relevant genotype	LD_99_ to MMS^a^ in mM	Relevant genotype	LD_99_ to MMS^a^ in mM	Impairment
*rec*^+^	41.2	Δ*rarA*	>50	

Δ*addAB*	0.8	Δ*addAB* Δ*rarA*	44.0	
Δ*recJ*	2.2	Δ*recJ* Δ*rarA*	4.6	End
Δ*recQ*	2.4	Δ*recQ* Δ*rarA*	4.7	Resection
Δ*recS*	2.3	Δ*recS* Δ*rarA*	4.8	

Δ*recU*	1.7	Δ*recU* Δ*rarA*	21.3	
Δ*recG*	2.2	Δ*recG* Δ*rarA*	4.8	Processing
Δ*ruvAB*	4.0	Δ*ruvAB* Δ*rarA*	5.0	Recombination
Δ*radA*	17.1	Δ*radA* Δ*rarA*	36.8	Intermediates

Δ*recO*	0.6	Δ*recO* Δ*rarA*	0.9	RecA
*recF15*	0.7	*recF15* Δ*rarA*	0.8	Accessory
Δ*recX*	10.6	Δ*recX* Δ*rarA*	7.6	Proteins

Δ*recD2*	36.6	Δ*recD2* Δ*rarA*	43.0	Undefined

*^*a*^The acute lethal dose of MMS that reduced cells survival by 99% (LD_99_) upon 15 min exposure.*

### Cell Staining

The LIVE/DEAD BacLight bacterial viability kit was purchased from Fisher Scientific. Cells were exponentially grown in NB to an OD_560_ = 0.4 at 37°C with agitation for 30 min. When indicated 3 μM MMC was added. Appropriate dilutions were stained with membrane-permeant SYTO 9, which labels living bacteria with green fluorescence, and with membrane-impermeant propidium iodide (PI), which enters and stains cells with red fluorescence. When cells are permeant to PI, its counterstaining activity competes with SYTO 9 for binding to DNA, and SYTO 9 staining signal is not detected. Red and green cells were counted using a fluorescence microscope and appropriate filters (470 ± 20 nm excitation filter and 515 ± 20 nm emission filter for both SYTO 9 and PI), as reported (Sanchez et al., 2007). In each experiment > 1000 cells were counted.

### RecA Protein Quantification

For quantification of RecA, quantitative Western blots were performed. Cells were grown in NB to an OD_560_ = 0.4 at 37°C with agitation and treated with increasing MMC concentrations (0.07–1.5 μM) for 30 min to induce the *recA* gene, which is under the control of a lexA regulated promoter (SOS induction). Cells (2 ml) were centrifuged, resuspended in 100 μl of buffer A (50 mM Tris HCl, pH 7.5, 1 mM DTT, 5% glycerol) containing 300 mM NaCl and lysed by sonication. Extracts from each experimental condition, containing similar concentrations of total and housekeeping proteins, were separated on 10% sodium dodecyl sulfate (SDS)-polyacrylamide gel electrophoresis (PAGE) alongside the purified RecA protein standard (10–500 ng) as reported (Cárdenas et al., 2012). Gels were transferred, and Western blots were developed with rabbit polyclonal anti-RecA antibodies (Cárdenas et al., 2012). This antibody showed no signal in the absence of RecA, suggesting that no cross-reactive signal interferred in our studies.

RecA protein bands on developed immunoblots were quantified with a scanning densitometer (ImageLab software, BioRad). Purified RecA protein standard yielded a linear relationship between antibody signal and the RecA protein concentration. The amount of RecA protein in each induced sample was interpolated from the standard curve performed with known amounts of purified protein, as described previously (Cárdenas et al., 2012). The *in vivo* concentration of RecA was estimated considering the cell volume of 1.2 femtoliters, and the amounts of cells loaded in the gel, based on the total number of CFUs.

### RecA ATP Hydrolysis Assays

The ssDNA-dependent ATP hydrolysis activity of RecA protein was assayed via a coupled spectrophotometric enzyme assay as described (Yadav et al., 2012) in buffer B (50 mM Tris-HCl pH 7.5, 1 mM DTT, 80 mM NaCl, 10 mM magnesium acetate, 50 μg/ml BSA, 5% glycerol) containing 5 mM ATP (30 min, 37°C). An ATP regeneration system (0.5 mM phosphoenolpyruvate, 10 units/ml pyruvate kinase) and a coupling system (0.25 mM NADH, 10 units/ml lactate dehydrogenase) were also included (Yadav et al., 2012). The order of addition of 3,199-nt pGEM3 Zf(+) ssDNA (10 μM in nt) and of the purified proteins is indicated in the text. Proteins used were RecA (650 nM), RarA (50 nM) and RarA K51A (100 nM) and SsbA (150 nM) that were purified as early described (Manfredi et al., 2008; Carrasco et al., 2018). Data from ATP hydrolysis were converted to ADP and plotted as a function of time, as described (Yadav et al., 2012).

### Fluorescence Microscopy and Data Analysis

A C-terminal fusion of the fluorescent protein mVenus to RecA was generated by cloning the 3′-end 500-bp of *recA* (excluding the stop codon) into plasmid pSG1164 mVenus (Lucena et al., 2018), which was integrated into the *recA* gene locus on the *B. subtilis* chromosome by single crossover recombination. Epifluorescence microscopy was used to monitor filament formation and dynamics of RecA before and after stress conditions at 30°C (OD_600_ = ∼0.3). Cells were treated with 0.5 mM H_2_O_2_ (obtained from Sigma Aldrich) or were not treated. For fluorescence microscopy, *B. subtilis* cells were grown in S7_50_ minimal medium at 30°C under shaking conditions until exponential growth, using a Zeiss Observer Z1 (Carl Zeiss) with an oil immersion objective (100 × magnification, NA 1.45 alpha Plan-FLUAR) and a CCD camera (CoolSNAP EZ, Photometrics). Electronic data were processed using Metamorph 7.5.5.0 software (Molecular Devices, Sunnyvale, CA, United States), which also allows the calibration of the fluorescence intensity and pixel size to determine the cell length and BacStalk (Hartmann et al., 2018). Time-lapse epifluorescence microscopy images of RecA-mV were collected every 5 min.

## Results and Discussion

### Experimental System

HR is the ultimate step for error-free repair of a double strand break (DSB) and for promoting the re-establishment of replication forks during vegetative growth. Many of the functions required for HR are conserved among bacteria. The *B. subtilis recA*, *recF*, *recO*, *recG*, *recJ*, *recQ, recR*, *ruvA*, *ruvB*, *radA*, and *rarA* genes have their counterpart in *E. coli* genes with identical name, whereas the *addAB* and *recU* genes have their counterpart in *E. coli recBCD* and *ruvC.* The *recS* and *recD*2 genes are absent in *E. coli* (Ayora et al., 2011; Alonso et al., 2013), and in contrast, *B. subtilis* cells lack *E. coli* ExoI (SbcB) and the RecA modulators DinI and RdgC (Cox, 2007).

The repair of a DSB by HR is a multistep and multiprotein process. Our current understanding of this process in *B. subtilis* is depicted in Figure 1. All these steps are conserved in bacteria and have been extensively reviewed both in *E. coli* and *B. subtilis* cells (Michel et al., 2001; Cox, 2007; Persky and Lovett, 2008; Ayora et al., 2011; Alonso et al., 2013; Kowalczykowski, 2015; Bell and Kowalczykowski, 2016). In short, repair by HR begins with DNA damage recognition by RecN, followed by the nucleolytic degradation of the 5′-terminated strands of a DSB by DNA end resection enzymes: the AddAB complex, or the RecJ-RecQ[RecS] complex in concert with SsbA (Figure 1a). The relevance of these two end resection pathways and their fine tuning differ between *E. coli* and *B. subtilis* cells (Persky and Lovett, 2008; Alonso et al., 2013; Kowalczykowski, 2015). In the presence of ATP, *B. subtilis* RecA cannot be loaded onto SsbA-coated ssDNA (Lovett and Roberts, 1985), and AddAB cannot activate RecA to catalyze DNA strand exchange (Carrasco et al., 2015). Therefore, once end resection functions generate a 3′-tailed duplex the RecA accessory proteins (known as mediators [SsbA, RecO, RecR]) recruit RecA onto SsbA-coated ssDNA (Figure 1b). The RecA modulators [RecF, RecX, RecU, and RarA, this work]) regulate RecA filament growth onto ssDNA, respectively (Figures 1b,c). The resulting RecA nucleoprotein filament, with the help of mediators and modulators drives homology search. Once found, the RecA nucleoprotein filament invades the homologous DNA, and dislodges one of the strands to form a three-strand recombination intermediate called a joint molecule or displacement loop (D-loop) (Figure 1d).

At the D-loop, the invaded strand primes DNA synthesis using the intact homologous chromosome (Figure 1e). Then the second resected end is captured with the help of RecA and RecO, more DNA synthesis restores the genetic material lost by resection at both ends, and with the help of branch migration translocases (RuvAB, RecG, RadA/Sms, and perhaps RecD2) the intermediate migrates (Figure 1f). Alternative mechanisms may process these intermediates. The capture of the second end can lead to formation of double HJs, which can be resolved to generate crossover (CO) or non-crossover (NCO) products with the help of the branch DNA translocases (RuvAB and perhaps RecG) and the RecU HJ resolvase (Figure 1g). Alternatively, they can be dissolved by the concert action of RecQ-like DNA helicases, SsbA and a Type I DNA Topoisomerase to generate NCO products (Figure 1h). At present the action of the RuvAB translocase in concert with the RecU HJ resolvase has been *in vitro* reconstituted (Cañas et al., 2014; Suzuki et al., 2014). The recombination intermediate can be also disrupted from the first processed DNA end by poorly characterized branch migration translocases, and this end can anneal with the other resected end of the break (Figures 1i–k). This process, termed synthesis-dependent strand annealing (SDSA), generates NCO and it is poorly understood in *B. subtilis* cells.

To gain further insight into the involvement of RarA in repair-by-recombination, the Δ*rarA* mutation was moved into *rec*-mutant strains deficient in (i) DNA end resection (*addAB*, *recQ*, *recS*, *recJ*), (ii) RecA mediators (*recO*) and/or modulators (*recF*, *recX*, *recU*), and (iii) HJ processing and cleavage/dissolution (*recG*, *ruvAB*, *radA*, *recD*2, *recU*, *recQ*, *recS*) (see Supplementary Table S1). The mutant strains were exposed to DNA damaging agents for 15 min in NB medium. The MMS and H_2_O_2_ drugs were chosen for our analysis (further explanation in Supplementary Annex S1). Our previous work showed that RarA single mutants are very sensitive to H_2_O_2_-induced lesions, but in the absence of RarA, cells remain recombination proficient and apparently are as capable of repairing MMS-induced DNA lesions as *wt* cells (Romero et al., 2019b), showing that RarA deals differently with the effect of the two drugs.

We classified the different outcomes into “moderately sensitive” when the viability was reduced less than 10^2^-fold, into “sensitive” when it was reduced less than 10^3^-fold, into “very sensitive” when viability was reduced from more than 10^3^-fold and up to 10^5^-fold, and when the viability was reduced more than 10^5^-fold the mutant strain was considered “extremely sensitive” to the damaging agent.

### Δ*rarA* Reduces Viability in Δ*recO* and Δ*recA* in the Absence of DNA Damage Inducing Agents

**FIGURE 2:**
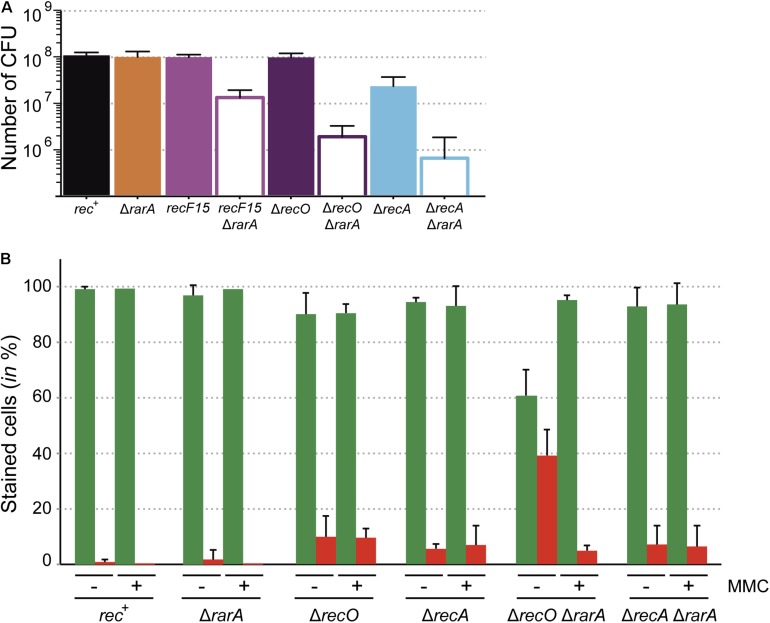
Growth defects of the Δ*rarA recF15*, Δ*rarA* Δ*recO* and Δ*rarA* Δ*recA* strains. **(A)** Cells were grown in NB to reach exponential phase (OD_560_ = 0.4) serially diluted, plated on NB agar, incubated ON and counted as CFU. **(B)** Cells were grown in NB to reach exponential phase (OD_560_ = 0.4) and divided in two aliquots (lacking [–] and containing 3 μM MMC [+]). The cultures were incubated for further 30 min and then cells were stained with SYTO 9 (green bar) and PI (red bar) to count the number of live and dead cells respectively. Percentage of SYTO 9- and PI-stained cells are indicated. 100% corresponds to the sum of green and red cells. The results are the average of at least three independent experiments and standard errors of the mean are indicated.

First we analyzed if viability is compromised when a Δ*rarA* mutation is combined with mutations in *rec*-proteins involved in DNA end resection (*addAB*, *recQ*, *recS*, *recJ*), RecA mediators (*recO*) and/or modulators (*recF*, *recX*, *recU*), and in HJ processing and cleavage/dissolution (*recG*, *ruvAB*, *radA*, *recD*2, *recU*, *recQ*, *recS*). In the absence of any external DNA damage the viability of the single mutant strains is not compromised, except in *recA*, *recG*, *ruvAB*, and *recU* mutants, which have a 5 to 10-fold reduction (Supplementary Figure S1 and Figure 2A) (Alonso et al., 1991; Cárdenas et al., 2012; Gándara and Alonso, 2015; Torres et al., 2017). Interestingly, in the absence of any external DNA damage the viability of the constructed strains listed in Supplementary Table S1 was quite different. The combination of Δ*rarA* with Δ*addAB*, Δ*recS*, Δ*recQ* or Δ*recJ* (impaired in alternative end resection pathways), Δ*recX* (negative modulator) or Δ*recD2* (a putative branch migration translocase) yielded similar or only slightly reduced (<1.4-fold) viability relative to *rec*^+^ cells (Supplementary Figure S1). Similarly, Δ*rarA* Δ*polY1* and Δ*rarA* Δ*polY2* double mutants have a viability similar than the single mutant strains (Romero et al., 2019b).

The deletion of *rarA* did not cause an extra fitness cost when combined with mutations in Δ*recU*, Δ*recG* or Δ*ruvAB*, and cell viability was not further reduced in these mutants (Supplementary Figure S1). The viability of the Δ*radA* cells was similar to or slightly reduced (<1.5-fold) relative to *rec*^+^ cells, but the viability of the Δ*rarA* Δ*radA* double mutant strain was reduced ∼10-fold compared to the *rec*^+^ strain (Supplementary Figure S1), suggesting that absence of the RarA and RadA/Sms functions poses a considerable threat for cell viability.

Previous studies demonstrated that in the absence of any external DNA damage, the Δ*recA* mutation leads to a strong reduction in cell viability in the absence of external DNA damaging agents (∼10-fold) (Carrasco et al., 2004; Romero et al., 2019b), whereas mutations in mediators and modulators as *recF*15 or Δ*recO* did not cause a reduction in cell viability (Figure 1a). Interestingly, the absence of RarA caused a ∼15-, ∼60-, and ∼145-fold reduction in the number of CFUs at mid-exponential phase in the Δ*rarA recF*15, Δ*rarA* Δ*recO* or Δ*rarA* Δ*recA* backgrounds, respectively, compared to the Δ*rarA* single mutant strain (Figure 2A). Thus, there is a strong synergic defect when combining the Δ*rarA* deletion with loss-of function in RecA accessory proteins or most severely with loss of RecA itself. Similarly, the *E. coli* Δ*rarA* Δ*recA* cells have low viability when compared to Δ*recA* cells (Shibata et al., 2005), revealing a strong parallel in this aspect.

Moving on with our analyses, we chose the double mutant strains with the lowest viability (Δ*recO* Δ*rarA* and Δ*recA* Δ*rarA*), to investigate whether this reduced viability correlates with membrane-compromised cells. Two different fluorophores were used (SYTO 9 and PI), which stain membrane-intact and membrane-compromised cells, respectively. Exponentially grown cells (OD_560_ = 0.4) were stained with SYTO 9 (in green) and PI (in red), and cells were analyzed by fluorescence microscopy. The proportion of exponentially growing *rec*^+^ and Δ*rarA* cells stained with PI (membrane compromised/dead) was low (∼1% and ∼1.8% of total cells, respectively). The proportion of Δ*recO* and Δ*recA* cells stained with PI was 9.8% and 5.6% of total cells, respectively (Figure 2B). The absence of RarA increased the proportion of PI stained cells by only ∼1.2 fold in Δ*recA* cells, but this number increased by ∼4-fold in the Δ*recO* background (Figure 2B). Thus, the strong decrease in CFUs in Δ*recA* Δ*rarA* cells (∼145-fold) does not correlate with the number of membrane compromised cells (7.1% of total cells), but it partially does in Δ*recO* Δ*rarA* cells (∼60-fold reduction in CFUs versus 39.2% PI staining cells) (Figure 2B). At present it is unknown whether the poor viability of Δ*recO* Δ*rarA* or Δ*recA* Δ*rarA* cells correlates with improper timing of the DNA damage response and/or the accumulation of toxic intermediates concurrent with DNA replication. RecO and RecA play a crucial and essential role, respectively, in repair-by-recombination and in the response to DNA damage (Gassel and Alonso, 1989; Goranov et al., 2006; Cárdenas et al., 2014). In addition, they are important for accurate ongoing DNA replication, being involved in the restart of stalled replication forks. Previous studies demonstrated that a transient block in cell proliferation (e.g., by chloramphenicol or rifampicin addition) renders *recO* mutants ∼100-fold more resistant to different DNA-damaging agent than the same dosis to proliferating *recO*^+^ cells (Carrasco et al., 2004). We therefore hypothesized that if DNA replication is halted the number of live *recO* or *recA* cells (i.e., stained with SYTO 9) might be recovered.

To test whether a DNA damage, which halts DNA replication, overcomes the PI staining, the single and double mutant strains were exposed to 3 μM MMC for 30 min to produce a DNA replication block and the maximal response to DNA damage (Sassanfar and Roberts, 1990; Goranov et al., 2006; Cárdenas et al., 2014). Then, cells were stained with SYTO 9 and PI, and quantified by fluorescence microscopy. In the presence of 3 μM MMC, the total number of PI-stained cells did not significantly change in the single mutants and in the *rec*^+^ control when compared with the values obtained in the absence of MMC (Figure 2B). Upon MMC addition, the number of SYTO 9 stained Δ*recO* Δ*rarA* cells was increased ∼6-fold, with a subsequent decrease of PI stained cells. This rescue effect was not observed in Δ*recA* Δ*rarA* cells (Figure 2B). All these results suggest that accumulation of PI stained cells is concurrent with defects in DNA replication in these mutants, as is induction of the SOS response (Simmons et al., 2007).

These results showed that Δ*recO* Δ*rarA* and Δ*recA* Δ*rarA* double mutant strains show a gross cell proliferation defect (Figure 2A) and that RecO is crucial to alleviate the membrane compromised defect (Figure 2B). In Δ*recO* Δ*rarA* such a defect is transiently suppressed upon halting DNA replication by MMC addition (Figure 2B). This suppression of PI-stained Δ*recO* Δ*rarA* cells upon inducing a replicative stress, suggest that cells are alive and metabolically active at growth arrest.

### RarA Is Not Required for End Resection but Affects the Outcome of Repair Events in End-Resection Mutants

**FIGURE 3:**
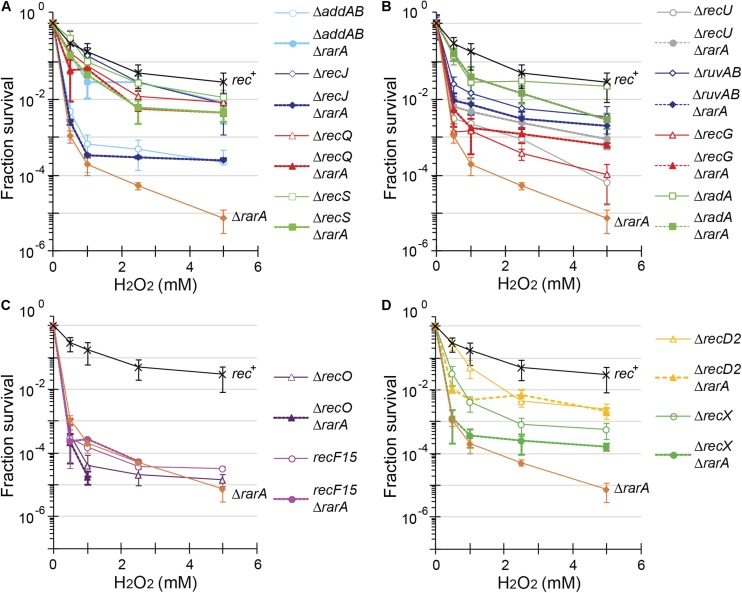
Acute viability assays of Δ*rarA* double mutant strains upon exposure to H_2_O_2_. Lack of RarA in cells impaired in end resection **(A)**, in processing of recombination intermediates **(B)**, in RecA accessory proteins **(C,D)** or in Δ*recD2* context **(D)**. Cells were grown to reach exponential phase (OD_560_ = 0.4), exposed to different concentrations of H_2_O_2_ for 15 min prior to serial dilutions. Cells were counted as CFU after ON growth, and results are plotted dividing these CFUs by the CFU obtained in untreated cells. The results are the average of at least three independent experiments and standard errors of the mean are indicated.

**FIGURE 4:**
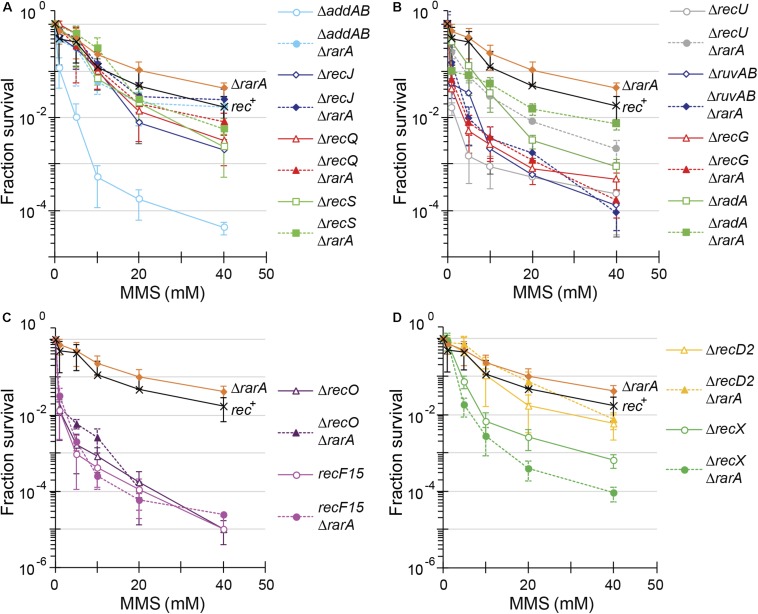
Acute viability assays of Δ*rarA* double mutant strains upon exposed to MMS. Lack of RarA in cells impaired in end resection **(A)**, processing of recombination intermediates **(B)**, in RecA accessory proteins **(C,D)** or lack of RecD2 **(D)**. Cells were grown to reach exponential phase (OD_560_ = 0.4), exposed to different concentrations of MMS for 15 min prior to serial dilutions. Cells were counted as CFU after ON growth, and results are plotted dividing these CFUs by the CFU obtained in untreated cells. The results are the average of at least three independent experiments and standard errors of the mean are indicated.

As described above, in *B. subtilis* there are two alternative DNA end resection pathways: the AddAB complex, and RecJ single-stranded exonuclease in concert with a RecQ-like DNA helicase (RecQ or RecS) (Figure 1a). The lack of both, AddAB and RecJ, renders cells extremely sensitive to DNA-damaging agents, with a sensitivity similar to that of Δ*recA* cells (Sanchez et al., 2006), showing that HR is no longer operative in their absence. In our experiments, Δ*addAB* mutations rendered cells very sensitive and the Δ*recS*, Δ*recQ* and Δ*recJ* mutations cells sensitive to H_2_O_2_ or MMS exposure (Figures 3A, 4A) (Sanchez et al., 2006), suggesting a certain hierarchical order in the processing of the broken molecules by the AddAB or RecJ-RecQ(RecS) complexes.

The acute lethal H_2_O_2_ dose that reduced Δ*rarA* cells survival by 99% (LD_99_) was ∼0.38 mM (Table 1), showing that the Δ*rarA* mutation rendered cells very sensitive to acute exposure to H_2_O_2_, with an LD_99_ > 16-fold lower than for the *rec*^+^ control (Figure 3 and Table 1) (Romero et al., 2019b). Curiously, the survival rate of Δ*addAB* Δ*rarA* cells was increased ∼12-fold when compared to the parental Δ*rarA* or Δ*addAB* strains (Figure 3A and Table 1), suggesting that in the absence of both RarA and AddAB the recombinational intermediates are channeled toward another repair pathway(s). The DNA repair defect of *rarA* mutant cells was also partially suppressed when the mutation was combined with *recQ* or *recS*, resulting in an LD_99_ to H_2_O_2_ that was ∼5-fold higher than that of Δ*rarA* cells (Figure 3A and Table 1). Thus, Δ*addAB*, Δ*recQ* or Δ*recS* mutations almost completely suppressed the DNA repair defect of the Δ*rarA* mutation upon exposure to H_2_O_2_.

The connection between *rarA* and *recJ* mutations was somewhat different than expected with regard to the above mentioned mutations. The survival rate of Δ*recJ* Δ*rarA* was reduced ∼9-fold compared to Δ*recJ*, and the LD_99_ was comparable to that of the Δ*rarA* control (Figure 3A and Table 1). At a higher H_2_O_2_ dose a different outcome was observed. At 2 mM H_2_O_2_ the survival rate increased ∼4-fold, and at 4 mM of H_2_O_2_ the survival of the Δ*recJ* Δ*rarA* mutant strain increased ∼17-fold compared to the Δ*rarA* control (Figure 3A), suggesting that the absence of *recJ* partially suppressed the DNA repair defect of Δ*rarA* cells at high H_2_O_2_ concentrations. The differences observed between the *recJ* and the other functions involved in end-processing in combination with Δ*rarA* could be due to the different activities. RecJ is involved in base excision repair, methyl-directed mismatch repair and HR (Persky and Lovett, 2008; Ayora et al., 2011; Bell and Kowalczykowski, 2016), whereas no role other than HR has been described for AddAB, RecQ or RecS (Ayora et al., 2011; Alonso et al., 2013). In none of the cases of double mutant cells we observed neither an epistatic effect, nor strong synergistic effects. Therefore, we have to assume that RarA is not required for end resection.

To further evaluate the contribution of RarA to end resection, exponentially growing cells were acutely exposed to increasing MMS concentrations for 15 min (Figure 4). The acute LD_99_ dose for MMS for *rec*^+^ cells (10 mM) was lower than that for Δ*rarA* cell (>50 mM) (Table 2), confirming that in the absence of RarA, cells remain recombination proficient, and apparently more capable of repairing MMS-induced DNA damage than *wt* cells (Romero et al., 2019b). AddAB cells were very sensitive to MMS, but the additional mutation in *rarA* rescued this phenotype: the LD_99_ to MMS was increased by ∼55-fold in Δ*addAB* Δ*rarA* cells relative to the Δ*addAB* mutant strain (Figure 4A and Table 2). The survival rate in Δ*recS* Δ*rarA*, Δ*recJ* Δ*rarA* or Δ*recQ* Δ*rarA* was enhanced ∼2-fold when compared to the single Δ*recS*, Δ*recJ* or Δ*recQ* strains (Figure 4A and Table 2). All these results show that in the absence of functions involved in long-range 5′→3′ end resection (e.g., AddAB, RecJ, RecQ, RecS) the DNA repair defect to MMS-and H2O2-induced lesions is partially suppressed in the Δ*rarA* context (Figures 3A, 4A), suggesting that in the absence of both RarA and an end resection pathway a new repair avenue is opened, or that RarA prevents uncontrolled DNA degradation by one of the two resection pathways, and inactivation of end-resection suppresses the need of RarA. This last hypothesis is in agreement with a previous report showing that WRNIP1 is directly involved in preventing uncontrolled MRE11-mediated degradation of stalled replication forks (Leuzzi et al., 2016). These genetic interactions are in line with the observation that exponentially growing Δ*addAB*, Δ*recS*, Δ*recQ* or Δ*recJ* cells show strongly reduced RarA-mVenus mobility (Romero et al., 2019a), *i.e.*, the activity of RarA with respect to its binding to DNA is considerably altered in end resection mutants.

### Branch Migration or HJ Processing of Recombination Intermediates Activities Do Not Require RarA, but Their Loss Partially Suppresses *rarA* Phenotypes

Bacterial RarA shares sequence homology with RuvB, a subunit of the RuvAB branch migration translocase (Barre et al., 2001). A branch migration translocase binds to HJs (formed as HR intermediates [double-HJ] or when replication forks stall and reverse [HJ-like structure]), and promotes branch migration (Higgins et al., 1976; Michel et al., 2001; Atkinson and McGlynn, 2009). Recent work has shown that RadA, which interacts with RecA, branch migrates recombination intermediates (Torres et al., 2019a). When its cognate site becomes available, the RecU resolvase cleaves the double HJ in concert with the RuvAB translocase, to preferentially generate NCO products, and rarely CO products (postsynaptic step) (Cañas et al., 2008; Atkinson and McGlynn, 2009; Ayora et al., 2011; Bell and Kowalczykowski, 2016). It is unknown whether RecU can cleave the reversed forks generated by RecG in *B. subtilis*. In any event, RecU has two activities: to mediate HJ cleavage in concert with a branch migration translocase (Cañas et al., 2014), and to modulate RecA nucleoprotein filament formation by its interaction with the RecA protein (Carrasco et al., 2005; Serrano et al., 2018).

In our assays, the Δ*recG*, Δ*ruvAB*, and Δ*recU* mutations rendered cells very sensitive and the Δ*radA* mutation sensitive to H_2_O_2_ or MMS exposure (Figures 3B, 4B) (Sanchez et al., 2005, 2007; Gándara et al., 2017; Torres et al., 2019b). The survival rate to H_2_O_2_ of Δ*radA* Δ*rarA* or Δ*ruvAB* Δ*rarA* mutant cells was increased compared to the less sensitive single mutant strain, with an LD_99_ to H_2_O_2_ ∼12-fold or ∼3-fold higher than the Δ*rarA* strain, respectively (Figure 3B and Table 1). The LD_99_ to H_2_O_2_ of the Δ*recG* Δ*rarA* or Δ*recU* Δ*rarA* mutant strains was similar to the more sensitive single mutant strain (Figure 3B and Table 1). However, at a H_2_O_2_ dose as high as 2 mM, the survival rate of Δ*recG* Δ*rarA* or Δ*recU* Δ*rarA* mutant strains increased ∼16-fold and ∼25-fold relative to the Δ*rarA* strain (Figure 3B), suggesting that Δ*recG* or Δ*recU* partially suppressed the DNA repair defect of Δ*rarA* cells at high H_2_O_2_ concentrations. When cells were acutely exposed to increasing MMS concentrations (Figure 4B), the sensitivity of Δ*recU* Δ*rarA*, Δ*recG* Δ*rarA* and Δ*radA* Δ*rarA* cells to MMS was lower than that of the single mutants, so that the LD_99_ to MMS was ∼ 2-, ∼2- and ∼12-fold higher than the Δ*radA*, Δ*recG* and Δ*recU* mutant strains, respectively, but the LD_99_ of the Δ*ruvAB* Δ*rarA* cells was similar to that of the Δ*ruvAB* strains (Figure 4B). At MMS doses as high as 20 mM, the survival rate of Δ*ruvAB* Δ*rarA* mutant strain increased ∼3-fold compared to the Δ*ruvAB* control (Figure 4B), suggesting that Δ*rarA* partially suppressed the DNA repair defect of Δ*ruvAB* cells at moderate MMS concentrations.

Taken together, it can be stated that (i) the absence of RuvAB, RecG, RadA/Sms or RecU partially suppressed the acute sensitivity to high H_2_O_2_ concentrations of Δ*rarA* cells (Figure 3B); (ii) the absence of RarA partially suppressed the repair defect seen in the absence of the branch migration translocase (RadA/Sms) or of the HJ resolvase (RecU) upon exposure to MMS (Figure 4B). This is consistent with the observation that in the absence of HJ-processing enzymes, the static RarA population decreases in *ruvAB*, *recG* and *radA* cells, meaning that RarA is less often bound to DNA, but increased in *recU* cells (Romero et al., 2019a), *i.e.*, RarA becomes more engaged with DNA in cells lacking RecU.

### RarA Is Epistatic to RecO and RecF in Response to DNA Damage

The two-component mediator SsbA and RecO (in conjunction with RecR), together with positive (RecF) and negative modulators (RecX, RecU), load RecA on a ssDNA gap or a 3′-tailed duplex ssDNA, regulate RecA filament growth, and activate RecA to catalyze DNA strand exchange (Figure 1b) (Kidane et al., 2004; Cárdenas et al., 2012; Lenhart et al., 2014; Le et al., 2017).

As previously shown (Alonso and Stiege, 1991; Fernández et al., 1999), *recF*15 and Δ*recO* cells are very sensitive to H_2_O_2_ or MMS exposure (Figures 3C, 4C). The double Δ*recO* Δ*rarA* or *recF15* Δ*rarA* mutant strains were equally sensitive to H_2_O_2_ or to MMS as the more sensitive single mutant strain, suggesting epistasis (Figures 3C, 4C and Tables 1, 2). This is consistent with the observation that *rarA* is epistatic to *recA* in response to H_2_O_2_- or MMS-induced DNA damage (Romero et al., 2019b). Moreover, the ratio of DNA bound to freely moving RarA-mVenus is altered in Δ*recO* or *recF*15 cells upon exposure to DNA damaging agents (Romero et al., 2019a), showing that the genetic interaction is reflected in the presumed activity of RarA. As described for *B. subtilis rarA* (Figures 3C, 4C), eukaryotic WRNIP1 functions in the same pathway as the Rad51 mediator BRCA2 (Leuzzi et al., 2016).

### Δ*rarA* Partially Suppresses the DNA Repair Defect of Δ*recD2* or Δ*recX* Cells Treated With H_2_O_2_

The negative modulator RecX has been shown to disassemble RecA nucleoprotein filaments (Cárdenas et al., 2012; Le et al., 2017), but little is known about RecD2, whose function in HR is poorly understood (Walsh et al., 2014; Torres et al., 2017). Investigating the genetic connection between RarA and RecX or RecD2, we found Δ*recX* and Δ*recD2* mutants to be sensitive to acute H_2_O_2_ or MMS exposure (Figures 3D, 4D), as described earlier (Cárdenas et al., 2012; Torres et al., 2017). The LD_99_ to H_2_O_2_ of the Δ*recD2* Δ*rarA* or Δ*recX* Δ*rarA* double mutant strain was not significantly different that the Δ*rarA* strain (Figure 3D and Table 1). However, at a H_2_O_2_ dose as high as 2 mM, the survival rate of Δ*recX* Δ*rarA* or Δ*recD2* Δ*rarA* mutant strain was increased ∼4-fold or ∼100-fold, respectively, compared to the Δ*rarA* control, suggesting that Δ*recX* and Δ*recD2* partially suppress the DNA repair defect in the Δ*rarA* context at high H_2_O_2_ concentrations. With respect to MMS treatment, the Δ*recD*2 mutation partially suppressed the DNA repair defect of Δ*recD*2 Δ*rarA* cells (Figure 4D and Table 2), whereas the Δ*recX* Δ*rarA* strain was slightly more sensitive to MMS than the single Δ*recX* mutant strain (Figure 4D and Table 2). Thus, while the *recX* and *recD2* deletions have suppressor phenotype to high H_2_O_2_ concentrations with regards to the *rarA* deletion, Δ*recX* Δ*rarA* cells show higher sensitivity to MMS treatment than the Δ*recX* control (Figure 4D). Interestingly, RarA-mVenus dynamics decreased in the Δ*recX* strain (RarA was more strongly bound to DNA than in *wt* cells), and the opposite behavior was observed in the Δ*recO* or *recF*15 backgrounds (Romero et al., 2019a). Thus, there is a strong connection between RecX and RarA in a genetic and cell biological aspect.

### The Threshold for Maximal RecA Levels After DNA Damage Is Increased in Δ*rarA* Cells

The previous results suggest that RarA has two roles: it may protect DNA from deleterious action of recombination proteins, and additionally it may work as a RecA accessory protein, together with the RecO mediator and the RecF modulator. *In vitro, B. subtilis* RecA⋅ATP cannot nucleate onto SsbA coated ssDNA, and cannot catalyze DNA strand exchange between circular ssDNA and linear duplex in the absence of accessory factors (Lovett and Roberts, 1985; Carrasco et al., 2008, 2015). Thus, RecA activity is regulated by accessory proteins (Cox, 2007).

Damages in the DNA template block DNA replication in a concentration dependent manner, leading to extended ssDNA regions coated by SsbA. *B. subtilis* RecA⋅ATP acts as a sensor of excessive ssDNA, and with the help of mediators, it assembles onto the SsbA-coated ssDNA to generate RecA^∗^ (a RecA⋅ATP nucleoprotein filament). When cells are treated with UV light these different dynamic RecA filaments (RecA^∗^) chaperone the LexA transcriptional repressor, and facilitate its auto-cleavage (Little, 1991), thereby de-repressing ∼33 genes (*recA* among them) (Au et al., 2005), and activating the SOS response (Friedberg et al., 2006). A more general RecA-dependent DNA damage response is triggered following MMC-induced replication arrest, with ∼140 genes showing altered expression, including LexA-dependent (*e.g., ruvA* gene) and LexA-independent (*e.g., recN* gene) genes (Goranov et al., 2006; Cárdenas et al., 2014). Increased RecA expression can be taken as an indirect, but sensitive, measurement of *in vivo* RecA nucleation and subsequent polymerization (RecA^∗^) (Cárdenas et al., 2012, 2014).

**FIGURE 5:**
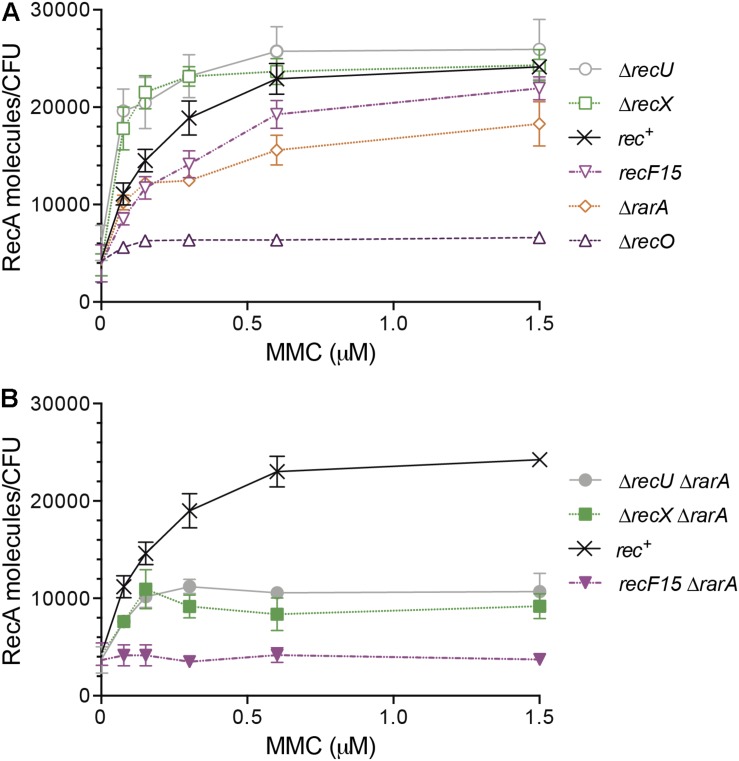
RecA protein accumulation upon SOS induction in different genetic backgrounds affecting RecA nucleoprotein dynamics. Exponentially grown *wt* (*rec*^+^), Δ*recX*, Δ*recU*, *recF*15, Δ*recO* and Δ*rarA* cells **(A)** or *wt*, Δ*recX* Δ*rarA*, Δ*recU* Δ*rarA* and *recF*15 Δ*rarA* cells **(B)** were exposed to the indicated concentrations of MMC for 30 min. Then cells were collected, lysed and equivalent protein amounts subjected to 10% SDS-PAGE, followed by immunoblot transfer. The number of RecA molecules/CFU are derived from a standard curve of known RecA concentrations and are the average of at least three independent experiments and standard errors of the mean are indicated.

Exponentially growing cells are estimated to contain ∼4,800 RecA monomers/CFU as judged by Western blot (Figure 5A) and by integrated mass spectrometry and 2-D gel-based proteomics analyses (Maass et al., 2011). In *rec*^+^ cells, there is a linear correlation between increasing MMC concentrations and the DNA damage threshold necessary to fully de-repress RecA expression. In *wt* cells, this RecA maximal level of expression is reached at ∼0.6 μM MMC, with a ∼5-fold increase, to 26,000 ± 1,000 RecA/CFU (Figure 5A), similar to what it was shown before in the *wt* as well as in the Δ*lexA* background (Cárdenas et al., 2012, 2014). Under similar experimental conditions, *recA* promoter utilization increased 6- to 10-fold (Gassel and Alonso, 1989). For comparison, undamaged *E. coli* cells have 7,000–15,000 RecA monomers/cell and these levels increase to ∼100,000 RecA/cell upon DNA damage (Boudsocq et al., 1997).

When cells were treated with H_2_O_2_, RecA reached its maximal level of expression at 3 mM H_2_O_2_, and its maximal induction was a ∼4-fold increase to 17,800 ± 1,050 RecA/CFU. However a linear correlation between RecA accumulation and H_2_O_2_ concentrations was less pronounced (Cárdenas et al., 2014), therefore MMC was used for further analyses.

Two different outcomes can be envisioned upon addition of increasing MMC concentrations in the absence of a RecA mediator or modulator. First, in the absence of a mediator or a positive modulator, negative RecA modulators present in the cell will promote a net RecA-ssDNA filament disassembly, with subsequent reduction in the probability of LexA repressor autocleavage. Thus, a higher MMC dose should be required to reach maximal RecA expression levels. Secondly, in the absence of negative modulators, the positive mediators and/or modulators will facilitate RecA-ssDNA filament assembly, so that the probabilities of RecA-ssDNA filament formation increase, as well as the interaction with LexA. Thus, a lower dose of DNA damage should be sufficient for RecA to stimulate LexA auto-cleavage, so maximal RecA levels are obtained at lower MMC doses in the absence of negative regulators. For example, in the absence of the positive modulator RecF, an MMC dose higher than the one needed in the *rec*^+^ control was required to have maximal RecA expression levels, but in the absence of negative modulator RecX, a lower MMC dose was sufficient (Figure 5A) (Cárdenas et al., 2012; Le et al., 2017).

We then tested whether RarA contributes to RecA nucleoprotein filament formation and compared its RecA levels with that in the absence of RecO (positive mediator) or RecF (positive modulator). In uninduced Δ*rarA*, Δ*recF*15 or Δ*recO* cells, RecA levels were maintained at a similar basal level estimated to be 4,600 ± 1,200 RecA monomers/CFU during mid-log phase of cell growth (Figure 5A). In the absence of RarA a full induction of *recA* was not observed, and maximal RecA levels lowered from ∼26,000 to 16,000 ± 900 RecA/CFU. These levels were reached at ∼0.75 μM MMC, and did not barely change at 1.5 μM MMC (Figure 5A). Similarly, a higher MMC dose is necessary to facilitate maximal RecA expression in cells impaired in the RecF modulator, but no SOS induction is observed in cells lacking RecO (Figure 5A) (Cárdenas et al., 2012). Because both, RarA and RecO, interact with SsbA (Costes et al., 2010), it is unlike that RarA binds to the RecA filament and competes with LexA binding, preventing its autocleavage. Thus, we can exclude this alternative explanation for a higher MMC dose required for maximal RecA expression levels, and proposed that RarA is a true mediator or modulator of RecA, and that it faciliates and/or stabilizes RecA filaments onto ssDNA.

### RarA Is Required for Efficient RecA Filament Formation *in vivo*

**FIGURE 6:**
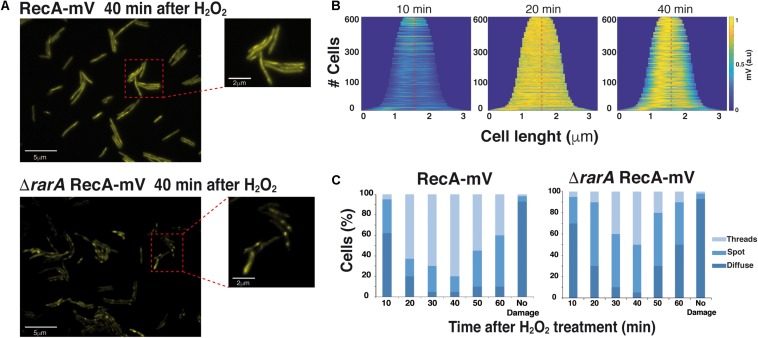
Epifluorescence microscopy showing that RecA assembly into threads is dependent on RarA. **(A)** Subcellular localization of RecA-mV 40 min after treatment with 0.5 mM H_2_O_2_, in *wt* (*rec*^+^) and in Δ*rarA* mutant cells. Scale bars 5 μm. **(B)** Demographs of *wt B. subtilis* cells, demonstrating the localization of RecA-mV to the central regions. Cells were aligned and ordered according to size. The fluorescence profiles represent the mean fluorescence values along the medial axis after background subtraction and normalization such that the maximum fluorescence of each cell is equal. **(C)** Quantitative analysis of RecA thread formation in *wt* or *rarA* mutant cells. The results are the average of three independent experiments (*n* = 450 cells).

To analyze whether RarA participates in RecA nucleation onto ssDNA and/or facilitates RecA-ssDNA filament growth, we used a functional RecA-mVenus (mVenus is a variant of fluorescent protein YFP), for the visualization of RecA filaments (termed “threads”) in live cells. This C-terminal fusion was integrated at the original gene locus, such that the fusion is the sole source of RecA expressed in cells, under the control of the original promoter. The RecA-mVenus fusion is repair proficient, as the RecA-mVenus strain was as viable as *wt* cells after induction of DNA damage, in contrast to the highly sensitive *recA* deletion strain. RecA-mVenus changed from a localization pattern throughout the cells (“diffuse”) or at discrete spots to form striking filamentous structures upon induction of DNA damage (Supplementary Figure S2). These filamentous structures have been described before (Kidane and Graumann, 2005b) and were termed “threads,” because it is still unclear if these structures correspond to RecA-ssDNA filaments observed *in vitro*. Although evidence for this notion have been described (Kidane et al., 2009; Lesterlin et al., 2014; Rajendram et al., 2015), we will maintain the term “threads” to describe the structures observed by epifluorescence microscopy. As control experiments, we imaged cells in the absence of induced DNA damage, where no filamentous structures are seen over a similar time frame (Supplementary Figure S2A), and imaged cells treated with H_2_O_2_, where a response very similar to that after MMC treatment was observed (Kidane and Graumann, 2005a). Formation of RecA threads was maximal 40 min after induction of DNA damage, and thereafter, threads dissipated in favor of the diffuse or spot-like localization seen in the absence of DNA damage (Supplementary Figure S2). Strikingly, even at 40 min after addition of H_2_O_2_, Δ*rarA* mutant cells only showed the RecA patch- or spot-like structures that occasionally had short filamentous extensions (Figure 6A). The failure to form discrete RecA threads can be most conveniently seen in the demographs (Figure 6B), which do not reflect different levels of RecA-mVenus, but visualize the presence or absence of sharply contrasted fluorescent structures, *i.e.*, RecA threads. In order to follow the dynamics of formation of RecA threads, we scored the number of cells containing diffusely localized RecA, RecA spots or RecA threads, during exponential growth (no damage) or in 10 min intervals following damage induction. Figure 6C shows that while less than 10% of exponentially growing cells contained visible RecA threads or spots (no damage), ∼65% of cells contained RecA threads and ∼15% RecA spots as early as 20 min after addition of H_2_O_2_, which declined thereafter back toward the pattern seen in untreated cells. In stark contrast, only a maximum of ∼15% of Δ*rarA* cells contained RecA threads, but ∼60% RecA spots only. Assuming that the accumulation of RecA into spots represents RecA loading events onto ssDNA, and the formation of threads extended filament formation, we can propose that RarA plays an important role in the formation of RecA threads by promoting the extension of filaments, stabilizing the RecA nucleoprotein filament or by downregulating the activity of negative modulators. Thus, RarA plays a dual role during HR: in addition to its activity in replication re-initiation (Carrasco et al., 2018), it also strongly affects the formation of RecA threads, which have been shown to be the active form of RecA during HR (Kidane and Graumann, 2005b).

### RarA Contributes to RecA^∗^ Accumulation

We can envision that RarA, RecO and RecF contribute to RecA^∗^ accumulation, but not RecX (Figure 5A), which *in vitro* acts as a negative modulator of RecA nucleoprotein filament formation as RecU (Le et al., 2017; Serrano et al., 2018). The role of RecU in RecA^∗^ accumulation *in vivo* is poorly understood, thus it was addressed. In the absence of MMC, RecA levels were estimated to be 4,600 ± 1,200 RecA monomers/CFU in Δ*recU* cells (Figure 5A). As expected for a negative modulator, a significant net RecA accumulation was observed upon exposure to low MMC concentrations in Δ*recU* cells. As low as 0.07 μM MMC already increased RecA levels, and the maximal level of RecA accumulation was reached at ∼0.3 μM MMC (26,000 ± 1,100 RecA/CFU) (Figure 5A). Similar results were observed in the absence of the negative modulator RecX (Figure 5A) (Cárdenas et al., 2012; Le et al., 2017).

To test whether RarA contributes to RecA^∗^ accumulation in a way that directly or indirectly it may antagonize the action of RecX or RecU, the expression levels of RecA were measured in Δ*recX* Δ*rarA* or Δ*recU* Δ*rarA* cells. The basal level of RecA in the Δ*recU*Δ*rarA* and Δ*recX* Δ*rarA* strains was slightly lower than in the *rec*^+^ cells (∼4,100 RecA monomers/CFU) (Figure 5B). In the presence of increasing MMC, RecA expression in Δ*recU*Δ*rarA* or Δ*recX* Δ*rarA* cells was similar to *rec*^+^ cells up to 0.15 μM MMC, but no further increase was observed at higher MMC concentrations (Figure 5B). These results suggest that the absence of RarA only partially counteracted the effect of the absence of RecU or RecX. The maximal levels of RecA accumulation were reduced in the double mutants: Δ*recU*Δ*rarA* reached 10,000 ± 1,200 RecA/CFU and Δ*recX* Δ*rarA* 8,400 ± 900 RecA/CFU (Figure 5B). This suggest that an unknown function(s) might fully counteract(s) the RecU or RecX activity. In the absence of RecU or RecX the requirement of RarA to fully induce the SOS response becomes essential, further confirming our conclusion that RarA facilitates RecA-ssDNA filament formation.

### RarA Acts as a Positive Contributor to RecA Filament Formation

Since RarA was epistatic with RecO and RecF upon DNA damage, but growth was reduced in Δ*recO*Δ*rarA* and *recF*15 Δ*rarA*, RecA expression levels after SOS induction were measured exposing cells to increasing MMC concentrations (Figure 5B). The RecA basal level of *recF15*Δ*rarA* cells was slightly lower than in the *rec*^+^ cells (∼4,100 ± 900 RecA monomers/CFU) (Figure 5B). In the double mutant background increasing concentrations of MMC failed to stimulate RecA expression (∼3,900 RecA/CFU) above the RecA basal levels (Figure 5B). This result suggested that RarA might work as a mediator or as an alternative positive modulator. In the absence of both RarA and RecF modulators, RecA could nucleate onto SsbA-coated ssDNA by the action of RecO, but these filaments are likely destabilized by RecX and/or RecU, so that no SOS induction is observed. Furthermore, these results are consistent with the observation that RecA forms foci, but the RecA threads are disassembled and become shorter in Δ*rarA* cells (Figure 6C).

The estimation of the RecA basal level in the Δ*recO*Δ*rarA* strain generated uncertainties (∼3200 ± 1900 RecA/estimated cell) due to the 60-fold lower viability of the Δ*recO*Δ*rarA* strain (see Figure 1b), and the high noise observed after MMC induction. Therefore, the strain was not further analyzed.

### RarA Might Stabilize a RecA Nucleoprotein Filament

*In vitro*, RecA⋅ATP can nucleate and polymerize on protein-free ssDNA, but RecA⋅ATP cannot nucleate or polymerize in the SsbA-ssDNA complexes (Carrasco et al., 2008, 2015), suggesting that RecA ATPase activity is inhibited in the presence of SsbA. The presence of the RecO mediator is necessary and sufficient to reverse the negative effect of SsbA on RecA nucleation and filament growth onto SsbA-coated ssDNA (Carrasco et al., 2008, 2015). The ATPase activity of RecA in the presence of the positive modulator RecF has been only studied in *E. coli* cells (Cox, 2007). Here, RecF marginally reduced the maximal rate of ATP hydrolysis by RecA ATPase activity when compared to RecA alone (Cox, 2007).

To characterize the role of RarA in RecA nucleation and/or polymerization onto ssDNA *in vitro*, we purified RecA, SsbA, the *wt* RarA protein, and the catalytically inactive Walker A mutant variant, RarA K51A (Carrasco et al., 2018), and used the kinetics of RecA-mediated ssDNA-dependent hydrolysis of ATP as an indirect readout of nucleation and filament growth (Manfredi et al., 2008).

**FIGURE 7:**
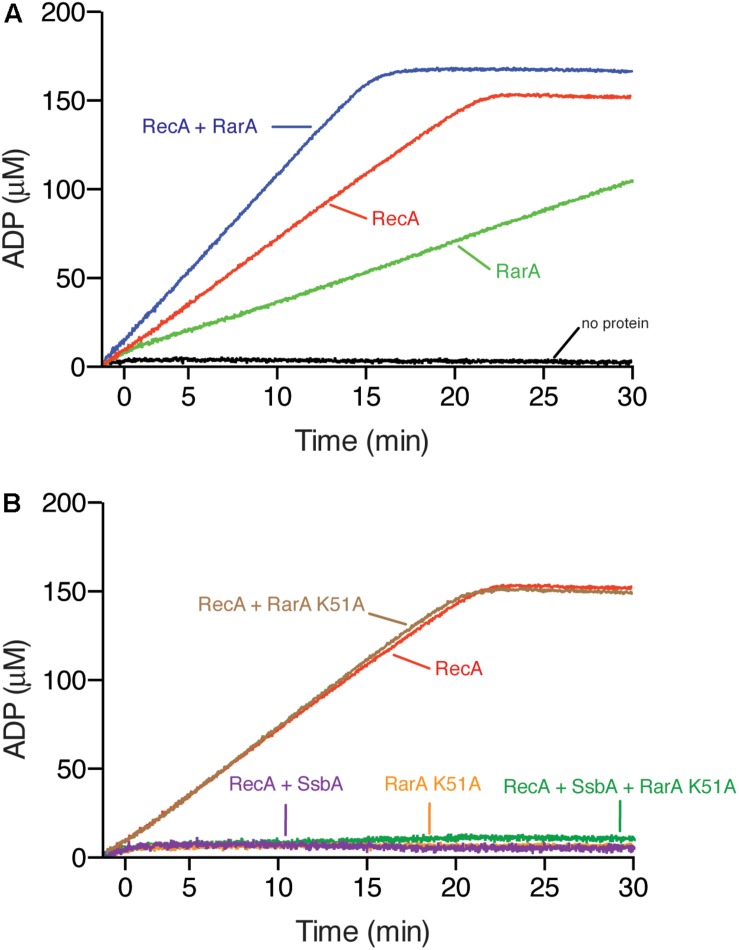
RarA effect on RecA nucleation and filament growth. **(A)** Circular 3,199-nt ssDNA (10 μM in nt) was incubated with RecA (650 nM), RarA (50 nM), RarA K51A (100 nM) or both RecA and RarA in buffer B containing 5 mM ATP and ATPase activity measured for 30 min. **(B)** Circular ssDNA (10 μM in nt) was pre-incubated with SsbA (150 nM) and RarA K51A (100 nM) in buffer A containing 5 mM ATP (5 min at 37°C), and then RecA was added or the ssDNA was pre-incubated with RarA K51A and RecA in buffer B containing 5 mM ATP (5 min at 37°C), and then SsbA was added and the ATPase activity measured for 30 min. The amount of ATP hydrolyzed was calculated. Representative graphics are shown here and quantification of the results are expressed as the mean ± SEM of >3 independent experiments.

In the presence of limiting RecA (650 nM, 1 RecA monomer/16 nucleotides [nt]), nucleation and polymerization on the 3,199-nt ssDNA showed a monophasic shape and ATP was hydrolyzed at a catalytic rate constant (K*_*cat*_*) of 9.3 ± 0.2 min^–1^ (Figure 7A), similar to data reported from comparable experimental conditions (Yadav et al., 2014; Carrasco et al., 2015). RarA also has a ssDNA-dependent ATPase activity. Under the experimental conditions 50 nM RarA (1 RarA tetramer/200-nt) quickly hydrolyzed ATP at a rate near to the formerly observed k_cat_ of 68.2 ± 0.2 min^–1^, but RarA K51A (1 RarA K51A tetramer/100-nt), which does not bind ATP, was unable to hydrolyze ATP (<0.1 min^–1^) (Figures 7A,B) (Carrasco et al., 2018).

Different outcomes are expected if RarA loads RecA onto SsbA-coated ssDNA or if it contributes to filament growth. First, if RarA activates RecA to nucleate onto SsbA-coated ssDNA as RecO does (Carrasco et al., 2015), addition of RarA to a pre-formed SsbA-ssDNA complex should recover the RecA ATPase activity. Second, it RarA stabilizes RecA onto ssDNA and facilitates its polymerization, but does not affect the dynamic behavior of RecA, then the ATPase activity should be higher than the sum of their independent activities. Finally, if RarA facilitates RecA stabilization onto ssDNA, but reduces the dynamic behavior of RecA the ATPase activity should be lower than the sum of their independent activities, as RecF*_*Eco*_* does (Cox, 2007).

First, we assayed ATPase activity in the absence of SsbA. RecA and RarA, at a RecA:RarA ratio of 13:1 were incubated with ssDNA (5 min at 37°C), then ATP was added and the ATPase activity was measured (30 min at 37°C). The maximal rate of ATP hydrolysis of the reaction with both proteins was significantly lower than the sum of their independent activities (k_*cat*_ 12.9 ± 0.3 min^–1^) (Figure 7A), suggesting that RarA stabilizes RecA on the ssDNA.

RarA K51A interacts with SsbA with similar efficiency that *wt* RarA (Carrasco et al., 2018). To test whether RarA can mediate RecA loading onto SsbA-coated ssDNA, and to analyse if RarA K51A stimulates the RecA-mediated ATP hydrolysis the ATPase activity was measured (30 min at 37°C). The maximal rate of ATP hydrolysis of RecA was marginally reduced (k_cat_ 8.8 ± 0.1 min^–1^) (Figure 7B), suggesting that the RarA K51A variant, unable to bind and hydrolyze ATP, directly or indirectly interacts with RecA.

In the presence of SsbA the ATPase activity of RarA is strongly stimulated and *wt* RarA interacts with SsbA with similar efficiency that RarA K51A (Carrasco et al., 2018), so that we could not analyze in this case loading of RecA onto SsbA-coated ssDNA measuring its ATPase activity in the presence of *wt* RarA. When ssDNA was pre-incubated with SsbA (150 nM) and RarA K51A (100 nM) (5 min at 37°C), then RecA and ATP were added and the ATPase activity was measured (30 min at 37°C). RecA under this condition cannot hydrolyze ATP (Figure 7B). Similar results were observed when ssDNA was pre-incubated with RarA K51A (100 nM) and RecA (5 min at 37°C), then SsbA and ATP was added (data not shown), suggesting that RarA K51A cannot load RecA onto SsbA-coated ssDNA.

## Conclusion

Genetic analyses reveal that RarA may act in the context of arrested replication forks in conjunction with a network of accessory proteins that affect the activity of the RecA recombinase (Figure 1). Our work indicates that RarA could prevent uncontrolled DNA end resection and processing of stalled replication forks by the branch migration translocases (Figures 3A,B, 4A,B).

Most importantly, we show that RarA positively regulates RecA filament formation, and directly or indirectly counteracts the role of the negative RecA modulators *in vivo* (Figure 1c). The *rarA* gene is epistatic to *recO* or *recF* in response to DNA damage, but *rarA* is not epistatic to *recX* in response to MMS-induced DNA damage (Figures 3B,D, 4B,D). These data are consistent with previous single molecule tracking experiments and suggesting that one of the RarA functions is related to RecA and its accessory proteins (Romero et al., 2019a). It has been proposed that dynamic interactions of RarA with RecO and RecF differ from those with RecX and RecU (Romero et al., 2019a). When DNA is damaged, the RecA threads persist for a longer time in the Δ*recX* cells (Cárdenas et al., 2012), but there is a reduced number of RecA threads in the Δ*rarA* cells (Figure 6C). Based on these findings, RarA K51A cannot promote RecA nucleation on the SsbA-ssDNA complex (Figure 7B), and we assumed that RarA might contribute to RecA polymerization onto ssDNA and RarA directly or indirectly might counteract the role of the negative modulators RecX and RecU that promote RecA filament disassembly. Preliminary biochemical results revealed that RarA even in the absence of ATP binding stabilizes a RecA nucleoprotein filament (Figures 7A,B), and indirectly may counteract the anti-RecA activity of PcrA (counterpart of UvrD*_*Eco*_*), as proposed in *E. coli* cells (Lestini and Michel, 2007). Our data are consistent with the observation that downregulation of FBH1 (a member of the conserved UvrD family), which is responsible for the removal of RAD51 (eukaryotic RecA homolog) from chromatin, can compensate for loss of WRNIP1 (eukaryotic RarA homolog) activity, reinforcing the hypothesis of a possible function of WRNIP1 in stabilizing RAD51 filaments upon a direct protein-protein interaction (Leuzzi et al., 2016).

Like eukaryotic WRNIP1 whose absence leads to extensive degradation of nascent DNA strands (Leuzzi et al., 2016), inactivation of *rarA* renders cells very sensitive to H_2_O_2_-induced lesion, but deletion of the major DNA end resection pathways partially suppresses the DNA repair defect (Figures 2A, 3A). Our data thus show that there are strong parallels between eu- and prokaryotic RarA-like proteins, and increase knowledge on the function of bacterial RarA at a molecular level. Together, our results highlight novel roles for RarA in HR which help to maintain replication fork integrity during normal growth and when forks encounter DNA damage.

## Data Availability Statement

All datasets generated for this study are included in the article/Supplementary Material.

## Author Contributions

HR, SA, PG, and JA designed the experiments. HR, ES, RH-T, BC, PC, SA, PG, and JA planned the experiments and interpreted the data. HR, ES, RH-T, BC, PC, and SA performed the experiments. HR, ES, RH-T, BC, SA, PG, and JA drafted the manuscript. SA, PG, and JA wrote the manuscript.

## Conflict of Interest

The authors declare that the research was conducted in the absence of any commercial or financial relationships that could be construed as a potential conflict of interest.
