# Supplementary material for: Bacillus subtilis RarA Acts as a Positive RecA Accessory Protein
Source: Front Microbiol. 2020 Feb 13;11:92. doi: 10.3389/fmicb.2020.00092 (PMC7031210; doi:10.3389/fmicb.2020.00092)
Supplement: Supplementary file 1 [file Data_Sheet_1.pdf]

Bacillus subtilis RarA acts as a positive RecA accessory protein

Hector Romero[1,2,3], Ester Serrano[1], Rogelio Hernández-Tamayo[2,3], Begoña Carrasco[1], Paula P. Cárdenas[1,†], Silvia Ayora[1], Peter L. Graumann[2,3*], Juan C. Alonso[1*]

[1]Department Microbial Biotechnology, Centro Nacional de Biotecnología, CNB-CSIC, 3 Darwin St., 28049 Cantoblanco, Madrid, Spain,

[2]SYNMIKRO, LOEWE-Zentrum für Synthetische Mikrobiologie, Hans-Meerwein-Straße, 35043 Marburg,

[3]Fachbereich Chemie, Hans-Meerwein-Straße 4, 35032 Marburg, Germany

Annex 1. Experimental approach for repair-by-recombination studies

To gain further insight into the involvement of RarA in repair-by-recombination the double (triple in case of Δ*rarA* Δ*addAB* or Δ*rarA* Δ*ruvAB*) mutant strains were exposed to DNA damaging agents, for 15 min, at concentrations that are bacteriostatic to *rec*$^+$ cells growing in nutrient broth (NB) medium. MMS and H_2O_2 were chosen, because both induce modifications in DNA bases, but in the presence of Fe(II), H_2O_2 treatment additionally generates DNA nicks (1). MMS- or H_2O_2-damaged bases are mainly repaired by direct DNA damage reversal, such as the guanine oxidation prevention/repair system, base excision repair or mismatch repair (1-3). Unrepaired MMS- or H_2O_2-lesions primarily halt elongation by the replicative DNA polymerase, and thereby stall replication fork progression. Stalled forks can be repaired by different repair-by-recombination or postreplication repair pathways (2-4). The H_2O_2 generated nicks collapse replication forks, and these intermediates can be repaired by different repair-by-recombination pathways (5-7).

Table S1. *Bacillus subtilis* strains used

Strains	Relevant genotype[a]	Source	Strains	Relevant genotype[a]	Source
BG214	*rec*$^+$	Lab. strain	BG1067	+ Δ*rarA*	(8)
BG190	+ Δ*recA*	(9)	BG1555	+ Δ*recA* Δ*rarA*	(8)
BG439	+ Δ*recO*	(10)	BG1433	+ Δ*recO* Δ*rarA*	(8)
BG129	+ *recF15*	(11)	BG1055	+ *recF15* Δ*rarA*	(8)
BG1455	+ Δ*recD2*	(12)	BG1421	+ Δ*recD2* Δ*rarA*	(8)
BG1065	+ Δ*recX*	(13)	BG1371	+ Δ*recX* Δ*rarA*	(8)
BG1337	+ Δ*addAB*	(14)	BG1107	+ Δ*addAB* Δ*rarA*	(8)
BG675	+ Δ*recJ*	(14)	BG1059	+ Δ*recJ* Δ*rarA*	(8)
BG705	+ Δ*recQ*	(14)	BG1575	+ Δ*recQ* Δ*rarA*	(8)
BG425	+ Δ*recS*	(14)	BG1563	+ Δ*recS* Δ*rarA*	(8)
BG855	+ Δ*recU*	(15)	BG1083	+ Δ*recU* Δ*rarA*	(8)
BG1131	+ Δ*recG*	(16)	BG1103	+ Δ*recG* Δ*rarA*	(8)
BG703	+ Δ*ruvAB*	(17)	BG1351	+ Δ*ruvAB* Δ*rarA*	(8)
BG1245	+ ΔradA	(18)	BG1373	+ Δ*radA* Δ*rarA*	(8)
PG5142	+ *recA-yfp*[b]	This work	PG5143	+ *recA-yfp* Δ*rarA*	This work

[a]All strains are derivatives of *B. subtilis* BG214 (*trpCE metA*5 *amyE1 ytsJ*1 *rsbV*37 *xre*1 *xkd*A1 *att*^{SPß} *att*$^{ICE\mathit{Bs}1}$). [b]RecA-mVenus is a variant of the monomeric RecA-Yfp protein.

References

1. T. Mahaseth and A. Kuzminov: Prompt repair of hydrogen peroxide-induced DNA lesions prevents catastrophic chromosomal fragmentation. *DNA Repair (Amst)*, 41, 42-53 (2016) doi:10.1016/j.dnarep.2016.03.012
2. B. Sedgwick: Repairing DNA-methylation damage. *Nat Rev Mol Cell Biol*, 5(2), 148-57 (2004) doi:10.1038/nrm1312
3. C. Lundin, M. North, K. Erixon, K. Walters, D. Jenssen, A. S. Goldman and T. Helleday: Methyl methanesulfonate (MMS) produces heat-labile DNA damage but no detectable in vivo DNA double-strand breaks. *Nucleic Acids Res*, 33(12), 3799-811 (2005) doi:10.1093/nar/gki681
4. K. H. Almeida and R. W. Sobol: A unified view of base excision repair: lesion-dependent protein complexes regulated by post-translational modification. *DNA Repair (Amst)*, 6(6), 695-711 (2007) doi:10.1016/j.dnarep.2007.01.009
5. A. Kuzminov: Collapse and repair of replication forks in *Escherichia coli*. *Mol Microbiol*, 16(3), 373-84 (1995)
6. N. S. Persky and S. T. Lovett: Mechanisms of recombination: lessons from *E. coli. Crit Rev Biochem Mol Biol*, 43(6), 347-70 (2008) doi:905610678 [pii] 10.1080/10409230802485358
7. S. Ayora, B. Carrasco, P. P. Cardenas, C. E. Cesar, C. Canas, T. Yadav, C. Marchisone and J. C. Alonso: Double-strand break repair in bacteria: a view from *Bacillus subtilis*. *FEMS Microbiol Rev*, 35(6), 1055-81 (2011) doi:10.1111/j.1574-6976.2011.00272.x
8. H. Romero, T. C. Rosch, R. Hernandez-Tamayo, D. Lucena, S. Ayora, J. C. Alonso and P. L. Graumann: Single molecule tracking reveals functions for RarA at replication forks but also independently from replication during DNA repair in *Bacillus subtilis*. *Sci Rep*, 9(1), 1997 (2019) doi:10.1038/s41598-018-38289-6
9. P. Ceglowski, G. Luder and J. C. Alonso: Genetic analysis of *recE* activities in *Bacillus subtilis*. *Mol Gen Genet*, 222(2-3), 441-5 (1990)
10. S. Fernández, Y. Kobayashi, N. Ogasawara and J. C. Alonso: Analysis of the *Bacillus subtilis* recO gene: RecO forms part of the RecFLOR function. *Mol Gen Genet*, 261(3), 567-73 (1999)
11. J. C. Alonso and A. C. Stiege: Molecular analysis of the *Bacillus subtilis recF* function. *Mol Gen Genet*, 228(3), 393-400 (1991)
12. R. Torres, H. Romero, V. Rodriguez-Cerrato and J. C. Alonso: Interplay between B*acillus subtilis* RecD2 and the RecG or RuvAB helicase in recombinational repair. *DNA Repair (Amst)*, 55, 40-46 (2017) doi:10.1016/j.dnarep.2017.05.004
13. P. P. Cárdenas, B. Carrasco, C. Defeu Soufo, C. E. Cesar, K. Herr, M. Kaufenstein, P. L. Graumann and J. C. Alonso: RecX facilitates homologous recombination by modulating RecA activities. *PLoS Genet*, 8(12), e1003126 (2012) doi:10.1371/journal.pgen.1003126
14. H. Sanchez, D. Kidane, M. C. Cozar, P. L. Graumann and J. C. Alonso: Recruitment of *Bacillus subtilis* RecN to DNA double-strand breaks in the absence of DNA end processing. *J Bacteriol*, 188(2), 353-60 (2006)
15. S. Fernandez, A. Sorokin and J. C. Alonso: Genetic recombination in *Bacillus subtilis* 168: effects of *recU* and *recS* mutations on DNA repair and homologous recombination. *J Bacteriol*, 180(13), 3405-9 (1998)
16. H. Sanchez, B. Carrasco, M. C. Cozar and J. C. Alonso: *Bacillus subtilis* RecG branch migration translocase is required for DNA repair and chromosomal segregation. *Mol Microbiol*, 65(4), 920-35 (2007) doi:MMI5835 [pii] 10.1111/j.1365-2958.2007.05835.x

17. H. Sanchez, D. Kidane, P. Reed, F. A. Curtis, M. C. Cozar, P. L. Graumann, G. J. Sharples and J. C. Alonso: The RuvAB branch migration translocase and RecU Holliday junction resolvase are required for double-stranded DNA break repair in *Bacillus subtilis*. *Genetics*, 171(3), 873-83 (2005) doi:genetics.105.045906 [pii] 10.1534/genetics.105.045906
18. C. Gándara and J. C. Alonso: DisA and c-di-AMP act at the intersection between DNA-damage response and stress homeostasis in exponentially growing *Bacillus subtilis* cells. *DNA Repair (Amst)*, 27, 1-8 (2015) doi:10.1016/j.dnarep.2014.12.007

Figures

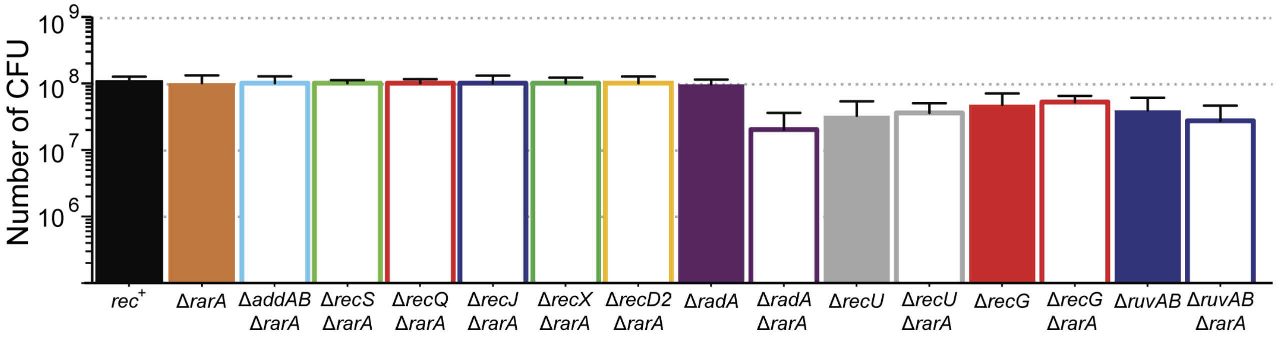

Figure S1. Growth defects of *rec*⁻ deficient strains in the Δ*rarA* context. Cells were grown in NB to reach exponential phase (OD_{560}=0.4) serially diluted, plated on NB agar, incubated ON and counted as CFU. The results are the average of at least three independent experiments and standard errors of the mean are indicated.

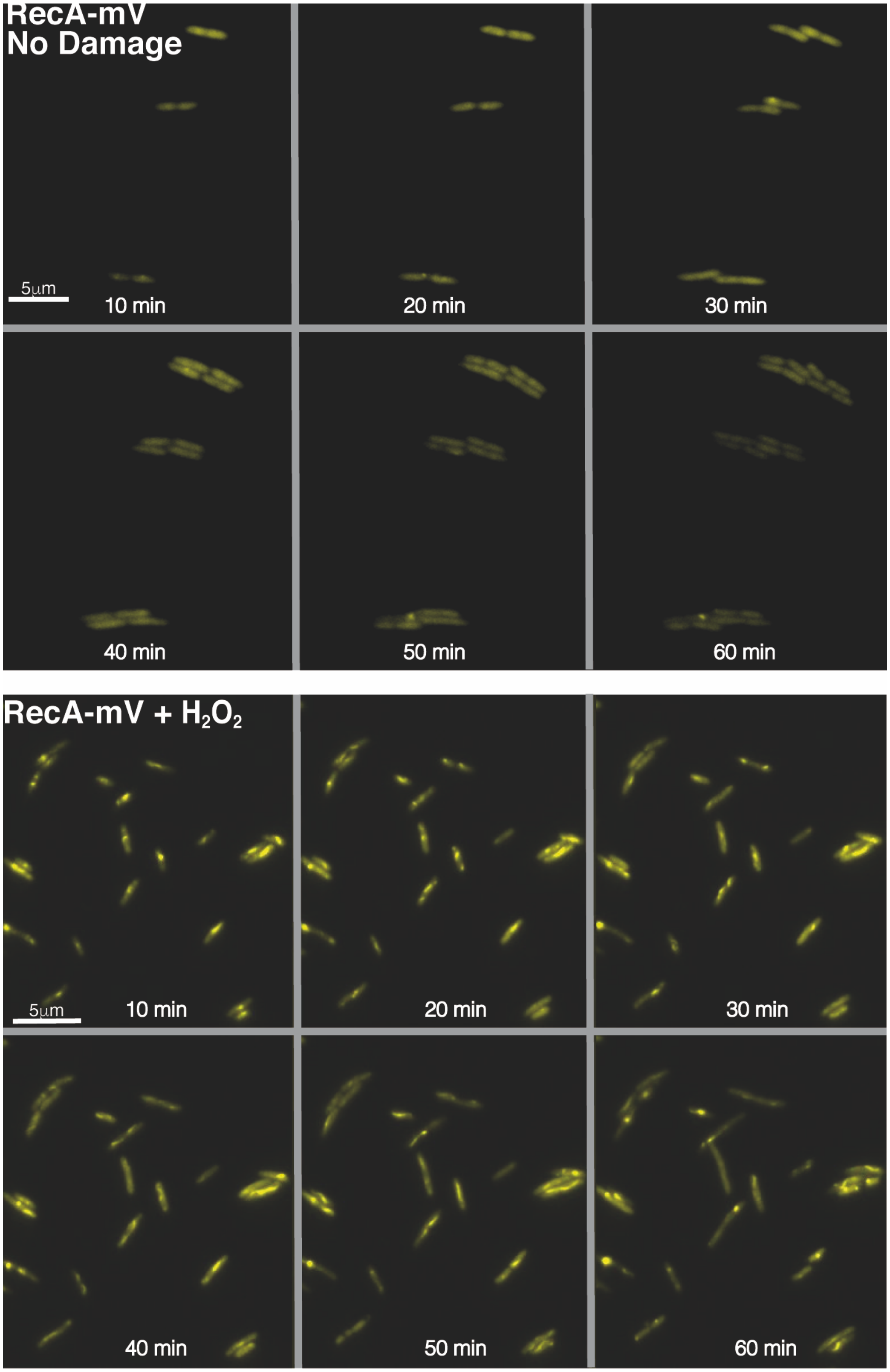

Figure S2. Time course of RecA assembly into discrete spots and extended filamentous structures called "threads" after H_2O_2 addition. Subcellular localization of RecA-mV after 10 min intervals in the absence (A) of the presence of 0.5 mM H_2O_2 treatment (B) in *rarA*$^+$ cells. Scale bars 5 µm.
